# Improvement on thermal performance of a disk-shaped miniature heat pipe with nanofluid

**DOI:** 10.1186/1556-276X-6-590

**Published:** 2011-11-14

**Authors:** Tsung-Han Tsai, Hsin-Tang Chien, Ping-Hei Chen

**Affiliations:** 1 Department of Mechanical Engineering, National Taiwan University, No. 1, Sec. 4, Roosevelt Rd., Taipei, 10617, Taiwan; 2 Microsystems Technology Division, Industry Technology Research Institute, No. 31 Gongye 2nd Rd., Annan District, Tainan, 70955, Taiwan

**Keywords:** heat pipe, heat spreader, electronic packaging, nanofluid

## Abstract

The present study aims to investigate the effect of suspended nanoparticles in base fluids, namely nanofluids, on the thermal resistance of a disk-shaped miniature heat pipe [DMHP]. In this study, two types of nanoparticles, gold and carbon, in aqueous solution are used respectively. An experimental system was set up to measure the thermal resistance of the DMHP with both nanofluids and deionized [DI] water as the working medium. The measured results show that the thermal resistance of DMHP varies with the charge volume and the type of working medium. At the same charge volume, a significant reduction in thermal resistance of DMHP can be found if nanofluid is used instead of DI water.

## Introduction

The demand for low cost and efficient cooling packaging has been increasing in recent years due to the large power density generated by electronic and optical devices. One of the choices is to use a heat pipe to spread the generated heat. A novel packaging base with a disk-shaped miniature heat pipe [DMHP] is proposed to replace the conventional copper base of the transmitter outline [TO] can package for a laser diode [1]. DMHP consists of multiple micro-grooves that radiate from the center of the base. The thermal performance of DMHP depends on the charge volume of the working fluid. It was found that the optimal volumetric fluid charge for the minimum thermal resistance is about 55%. In order to further increase the thermal performance of DMHP, a nanofluid was selected to replace deionized [DI] water as the working medium in the heat pipe.

Nanofluid has drawn the attention of researchers in the heat transfer community for heat transfer enhancement. Several previous studies showed that the thermal conductivity of a fluid could be significantly enhanced by adding suspended metal or nonmetal nanoparticles [2-6]. Xuan and Li [3] showed that the effective thermal conductivity of water-copper nanofluid is 75% greater than that of the base fluid (water in this case) even with only 8% volumetric fraction of particles in the base fluid. Besides, an experimental system was set up by Xuan and Li [7] to investigate the convective heat transfer phenomena of water-copper nanofluid in a tube. They found that the convective heat transfer coefficient in a tube could be increased by the addition of nanoparticles to the fluid when the volumetric fraction of the suspended nanoparticles was low.

Nanofluids have also been used in heat pipes in recent years [8-10], and the thermal enhancements of nanofluids on heat pipes were shown in these studies. There is no surprise that suspended particles in a fluid can affect the boiling heat transfer phenomenon at the solid-liquid interface. Huang et al. [11] showed that the pool boiling heat transfer of a heated stainless steel horizontal plate was significantly enhanced by adding glass, copper, and stainless steel microparticles into DI water. However, fluids with suspended microparticles may cause some problems such as abrasion and clogging [7]. Thus, they are not suitable for the applications of miniature heat pipes in which the pore size of the porous medium or the hydraulic diameter of the microchannel is of the order of the micrometer.

Therefore, the present study proposes to employ a nanofluid as a working medium of the DMHP. Two types of suspended nanoparticles were used, namely gold nanoparticles and carbon nanoparticles. A measuring system is also set up to investigate the effect of added nanoparticles in the fluid on the thermal resistance of DMHP.

## Preparation of nanoparticles

**Figure 1:**
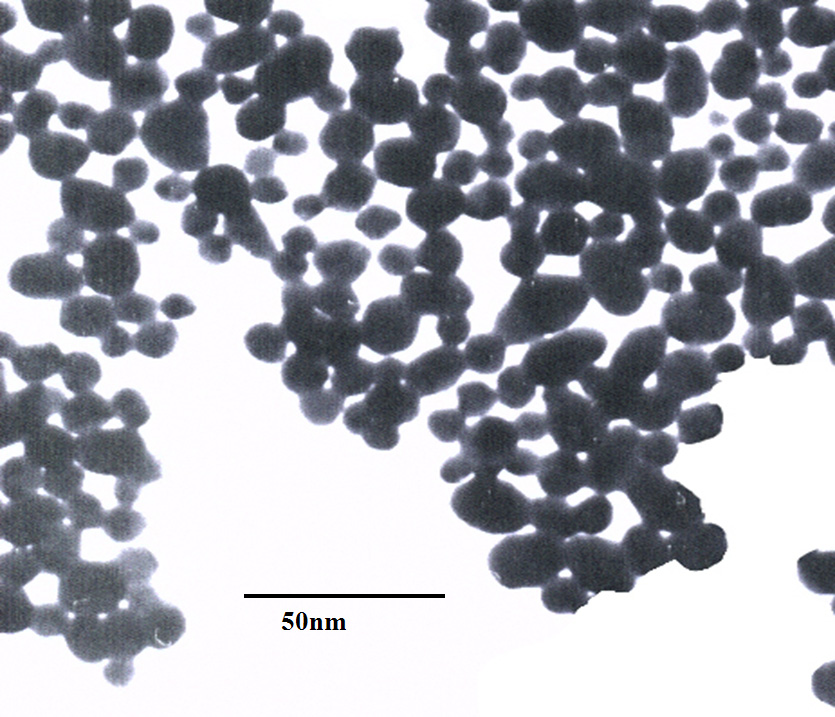
**TEM micrograph of gold nanoparticles with a magnification of 200,000**.

In the present study, gold nanoparticles were synthesized by citrate reduction from aqueous hydrogen tetrachloroaurate [HAuCl_4_] [12]. An amount of 0.008 g HAuCl_4 _(Sigma-Aldrich Chemical, St. Louis, MO) was dissolved in 80 ml distilled water as a primer solution. An additional 4-ml mixture of 3.4 mM (concentration of millimolar) citric acid, 0.1 ml of 5.8 mM tannic acid and 15.9 ml distilled water were used as a reducing solution. The reducing solution was preheated to 60°C. After the primer solution was heated to a boiling temperature, the reducing solution was then added into the primer solution. The mixed solution was stirred until the color of the mixed solution changed from transparent to red. The color change in the mixed solution indicated the formation of colloidal gold nanoparticles. Figure 1 shows a transmission electron microscope [TEM] (Hitachi 8100, Hitachi High-Tech, Minato-ku, Tokyo, Japan) micrograph of the gold nanoparticles with an average diameter of 17 nm; the volume fraction of the gold nanoparticles in the nanofluid was about 0.17%.

**Figure 2:**
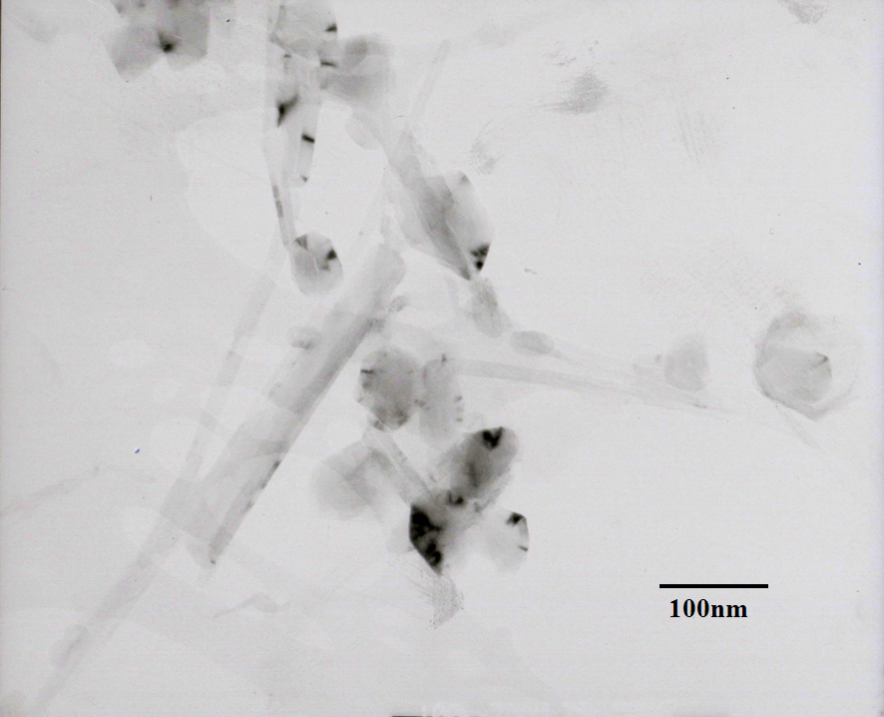
**TEM micrograph of carbon nanoparticles with a magnification of 100,000**.

There are several types of carbon nanoparticles. The most famous one is the so-called fullerene or C_60_. In this study, multiwall carbon nanoballs were used. They were prepared by arc discharge between graphite electrodes in reduced pressure of pure hydrogen gas. The carbon nanofluid used in this study is provided by Industrial Technology Research Institute of Taiwan. Figure 2 shows a TEM (Hitachi 8100, Hitachi High-Tech, Minato-ku, Tokyo, Japan) micrograph of carbon nanoparticles. As illustrated in Figure 2, multiwall carbon nanotubes and carbon nanoballs were produced at the same time during the fabrication process. They tend to aggregate together in the aqueous solution. The length of a multiwall carbon nanotube was over 200 nm, and the average diameter of a carbon nanoparticle was approximately 68 nm. For convenience, the mixture of multiwall carbon nanotubes and carbon nanoballs in the base fluid was still called carbon nanoparticles in this study. The volumetric fraction of carbon nanoparticles in the nanofluid was 9.7%.

## Measurements

**Figure 3:**
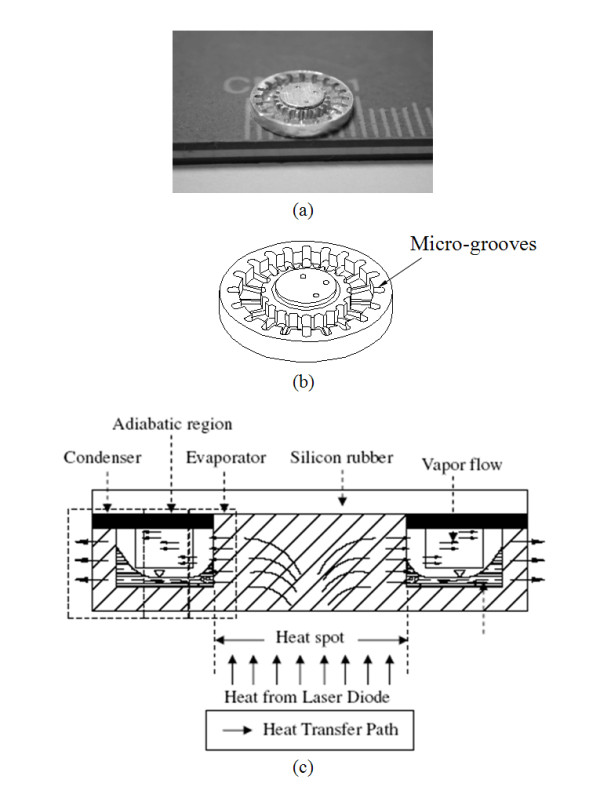
**The design of DMHP**. (**a**) A prototype, (**b**) three-dimensional view, and (**c**) the schematic plots of the evaporator, the adiabatic region, and the condenser [1].

Figures 3a and 3b, respectively, show a prototype and a three-dimensional view of the tested DMHP. Twenty micro-grooves were fabricated on an aluminum alloy (6061 T6) base by a precise metal forming process. These micro-grooves are evenly distributed. The diameter and thickness of the aluminum base are 9 mm and 2 mm, respectively. The depth and width of the micro-grooves are 0.4 mm and 0.35 mm, respectively.

Because the silicon rubber is elastic, it was used to seal the top of the aluminum base with vacuum grease and to keep the chamber airtight. An ultra-thin syringe needle was used to insert into the chamber and to pump the chamber down. Then, a syringe pumping controller is used to pump a proper quantity of working fluid into the chamber. For the present study, DI water and nanofluid at five different charges with 18%, 37%, 55%, 74%, and 92%, respectively, of the total void volume were used.

**Figure 4:**
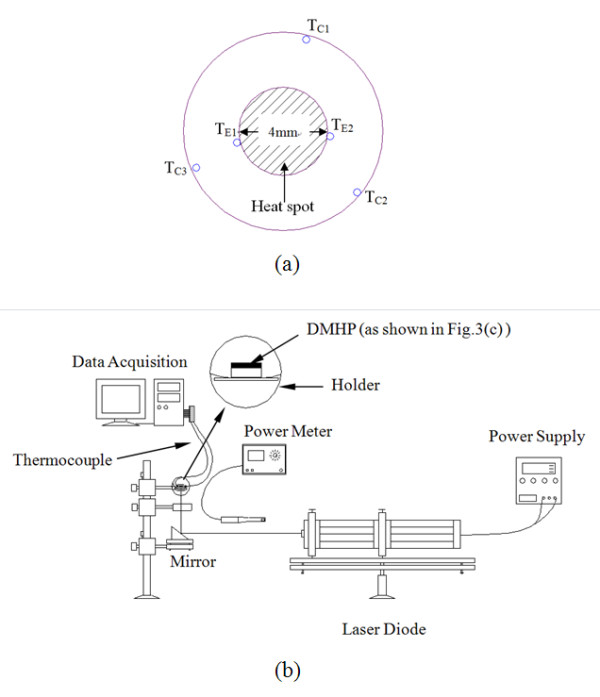
**Schematic diagram of the experimental setup**. (**a**) Distribution of the thermocouples and the heat spot and (**b**) the measuring system [1].

A schematic view of the apparatus for measuring the thermal performance of the DMHP is shown in Figure 3c. The tested DMHP was installed on the through hole of a Plexiglas holder. The Plexiglas holder with a through hole of 8.5 mm in diameter was positioned horizontally. The local temperatures on the DMHP surface were measured by five type T thermocouples. Some silicon heat transfer compounds are applied on the thermocouples. Then, the thermocouples are attached at the corresponding positions, and an annular silicon rubber is used to fix these thermocouples. Two thermocouples were attached to the center of the aluminum base plate to measure the evaporator temperature, and three were evenly distributed around the circumference to measure the condenser temperature. The distributions of the thermocouples are illustrated in Figure 4a. All thermocouples were calibrated against a quartz thermometer. The uncertainty in temperature measurement is about ± 0.1°C. The temperature of the evaporator was averaged by the two thermocouples beside the heat spot $\left(T_{\text{evap}}=\frac{T_{\text{E1}}+T_{\text{E2}}}{2}\right)$; and the temperature of the condenser was averaged by the other three thermocouples $\left(T_{\text{cond}}=\frac{T_{\text{C1}}+T_{\text{C2}}+T_{\text{C3}}}{3}\right)$.

A laser diode was used as the applied heat source in the measurement. The heating power of the laser diode was measured by an optical power meter (Vector H410, Scientech, Inc., Boulder, CO, USA) with a resolution of 0.001 W. The laser beam was focused on the center region (4 mm in diameter) of the aluminum base which was painted black with an aborptivity of *α*_λ _= 0.95. The applied heat loads were ranged from 0.1 to 0.6 W, and the heat fluxes were ranged from 4.7 to 28.2 KW/m^2^. Once both the heating load (*Q*) and the temperature difference (dT *= T*_evap _*- T*_cond_) were measured, the thermal resistance (*R*) could then be evaluated from the equation, *R = *dT/*Q*. The thermal resistance at each heat load could be calculated by the same process. The thermal resistances were averaged for all heat loads to be an averaged thermal resistance (*R*_av_) at each charge volume. The room temperature was kept at 20°C, and the measured temperature range is about 20°C to approximately 40°C. Based on the measurement error of the thermocouples and the power meter, the mean deviation of thermal resistance is about 13.9%.

For validation of basic properties of the working media, viscosity and thermal conductivity were measured. The viscosities of DI water and nanofluid were measured by a disk-type rotating viscometer (Brookfield RVTCP, Brookfield Engineering Lab., Middleboro, MA, USA). The uncertainty in viscosity measurement is about ± 3%. The thermal conductivity of DI water and nanofluid was measured by a transient hot wire method. The uncertainty in thermal conductivity measurement is about ± 2.3%.

## Results and discussion

**Figure 5:**
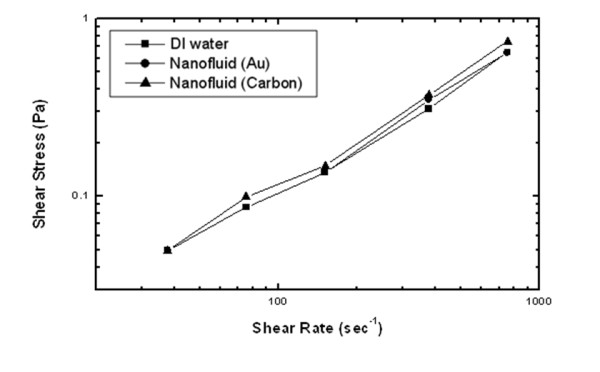
**Viscous properties of nanofluids and DI water**.

To characterize the flow properties of the nanofluid, the viscosity of the nanofluids was measured and compared with that of the DI water. Figure 5 shows the measured data between shear stress and shear rate for both nanofluids and DI water at 20°C. The results show that the relationships between shear stress and shear rate are almost linear for both nanofluids and DI water. This indicates that nanofluids with either gold nanoparticles or carbon nanoparticles are Newtonian fluids if the volumetric fraction of the nanoparticles in the base fluid is low. Table 1 lists the measured dynamic viscosities and thermal conductivities of nanofluids and DI water.

**Table 1:** Measured dynamic viscosities of nanofluid and DI water

Viscosity at 20°C	Viscosity measured in present study (mPa·s)	**Viscosity from Cengel**[13]**at 20°C (mPa·s)**	Thermal conductivity measured in the present study (W/mK)	**Thermal conductivity from Cengel **[13]**at 10°C (W/mK)**
Working fluid				
DI water	1.016	1.002	0.613	0.580
Nanofluid (Au nanoparticles)	1.036	-	0.67	-
Nanofluid (carbon nanoparticles)	1.125	-	0.68	-

The viscosity of DI water is almost the same as that in the data in the *Heat Transfer *textbook [13]. The data show that the viscosity of nanofluid with gold nanoparticles is close to that of DI water. Since the volume fraction of the gold nanoparticles is only 0.17% in this study, such a low concentration cannot have a large effect on the viscosity of the base fluid.

The present measured data show that the viscosity of the nanofluid with carbon nanoparticles is about 12% higher than that of the DI water. The volume fraction of carbon nanoparticles in the nanofluid is about 9.7%. As compared with the nanofluid with gold nanoparticles, the higher volume fraction of the carbon nanoparticles in the base fluid results in a greater viscosity of the nanofluid.

The measured values of the thermal conductivity of nanofluids and DI water are also listed in Table 1. The thermal conductivity of nanofluid with gold nanoparticles is only about 8.5% higher than that of DI water, which is within the uncertainty range of the measuring device. This increase in thermal conductivity with suspended gold nanoparticles is almost negligible when the volumetric fraction of nanoparticles in nanofluid is small. Based on the measured viscosity and thermal conductivity of the nanofluids, the physical properties of gold nanofluid are almost the same as those of DI water due to the low volumetric fraction of the nanoparticles in nanofluid.

**Figure 6:**
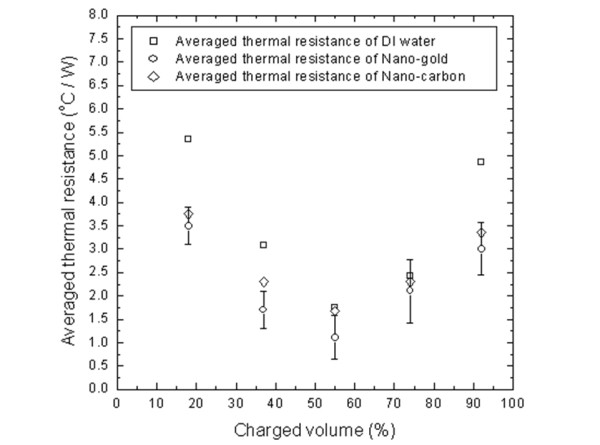
**Comparison on thermal resistances of DMHP for DI water and nanofluids under different charge volumes**.

Effects of the charge volume of all fluids on the thermal performance of tested DMHP are shown in Figure 6. The lowest thermal resistance occurs at a volumetric charge of 55% for all three tested fluids. For the clarity of the figure, only the error bars of the gold nanofluid are added. It is noted that the remaining two sets of error bars are in similar ranges with that of gold nanofluid. It is observed that, at the charge volumes of 18%, 37%, and 92%, the thermal resistances of DMHP with two nanofluids are much lower than those with pure water. At the charge volumes of 55% and 74%, the effect of charge volumes has a larger influence than that of the working fluid. Therefore, the reductions of thermal resistance of DMHP with two nanofluids are not very obvious, but they are still lower than those with pure water. It can also be observed that the thermal resistance of DMHP with a high volume fraction of carbon nanofluid is similar, even slightly higher than that with a low volume fraction of gold nanofluid. This may have resulted from the aggregation of carbon nanoparticles in a high volume fraction of nanofluid. Figure 6 also showed that the influence of the charge volumes on the thermal resistance of DMHP is more apparent than the effect of nanofluids.

Although the reductions of thermal resistances for nanofluids are not guaranteed for all charge volumes, the nanofluids somehow present a better thermal performance. There are several possible explanations for the enhanced heat transfer by the nanofluid. First, the nanofluids have larger convective heat transfer coefficients than those of pure fluids [7]. Second, the nanofluids have larger thermal conductivities than those of the pure fluids [3]. However, the above effects are only obvious for large volumetric fractions of the nanoparticles and not suitable for the present cases due to the low volumetric fractions. Xuan and Li [7] proposed one more possible explanation that the movement of nanoparticles improves the energy exchange process in the fluid. Tsai et al. [14] employed nanofluids as working mediums for a conventional circular heat pipe. Their results showed that the major reduction in the thermal resistance of the heat pipe is on the thermal resistance from the evaporator to the adiabatic section. The major thermal resistance occurring at the evaporator side is caused by the vapor bubble formation at the liquid-solid interface. Thus, the reduction of the thermal resistance may be related with the influence of nanofluid on the bubble formation at the evaporator side of the DMHP. The larger the nucleation size of a vapor bubble that will block the transfer of heat from the solid surface to the liquid, the higher the thermal resistance at the evaporator will be [14]. The suspended nanoparticles tend to bombard the vapor bubble during bubble formation. Therefore, it is expected that the nucleation size of a vapor bubble is much smaller for a fluid with suspended nanoparticles than that without them. Thus, a lower thermal resistance can occur at the solid-liquid interface for a fluid with suspended nanoparticles.

Due to the more uniform dispersion and smaller diameter of the gold nanoparticles in the base fluid, the gold nanofluid has a comparable thermal performance with carbon nanofluid of higher volume fraction.

## Summary and conclusions

The results showed that the dynamic viscosity of nanofluid with gold nanoparticles is close to that of DI water. The viscosity of nanofluid with carbon nanoparticles is 9% higher than that with gold nanoparticles.

As compared to a DMHP with DI water, the present measured data verify that the tested DMHP with gold nanoparticles and carbon nanoparticles do not have an obvious reduction of thermal resistance for all charge volumes. These are due to the low volumetric fraction of gold nanoparticles and the non-uniform dispersion and large diameter of carbon nanoparticles. It is also noted that the best charge volume is about 55% for all three working fluids.

For further enhancement of the thermal performance of the DMHP, the nanofluids of higher volumetric fraction and more uniform dispersion should be considered to be used as working fluids.

## Competing interests

The authors declare that they have no competing interests.

## Authors' contributions

PHC provided the idea and did the proofreading of the manuscript. THT drafted and revised the manuscript. HTC designed and carried out the experiment. All authors read and approved the final manuscript.

## References

[B1] Chien ST Lee DS Ding PP Chiu SL Chen PH Disk-shaped miniature heat pipe (DMHP) with radiating micro grooves for a TO can laser diode package IEEE Trans Comp Pack Tech 2003 26 569 574 10.1109/TCAPT.2003.817648

[B2] Wang BX Li H Peng XF Research on the heat-conduction enhancement for liquid with nano-particle suspensions J Therm Sci 2002 11 214 219 10.1007/s11630-002-0057-6

[B3] Xuan YM Li Q Heat transfer enhancement of nanofluids Int J Heat Fluid Flow 2000 21 58 64 10.1016/S0142-727X(99)00067-3

[B4] Wang BX Li H Peng XF A fractal model for predicting the effective thermal conductivity of liquid with suspension of nanoparticles Int J Heat Mass Tran 2003 46 2665 2672 10.1016/S0017-9310(03)00016-4

[B5] Choi SUS Enhancing thermal conductivity of fluids with nanoparticles ASME Fluids Eng Div 1995 231 99 105

[B6] Xuan YM Roetzel W Conceptions for heat transfer correlation of nanofuids Int J Heat Mass Tran 2000 43 3701 3707 10.1016/S0017-9310(99)00369-5

[B7] Xuan YM Li Q Investigation on convective heat transfer and flow features of nanofluids J Heat Tran 2003 125 151 155 10.1115/1.1532008

[B8] Wei WC Tsai SH Yang SY Kang SW Hassan I, Kobasko N Effect of nanofluid on heat pipe thermal performance Proceedings of the 3rd IASME/WSEAS Int Conf on Heat Transfer, Thermal Engineering and Environment: August 20-22, 2005; Corfu, Greece 2005 WSEAS Press 115 117

[B9] Wei WC Tsai SH Yang SY Kang SW Effect of nanofluid concentration on heat pipe thermal performance IASME Transactions 2005 2 1432 1439

[B10] Park KH Lee WH Lee KW Baek IH Rhi SH Shin DR Hassan I, Kobasko N Study on the operating characteristics in small size heat pipe using nanofluids Proceedings of the 3rd IASME/WSEAS Int Conf on Heat Transfer, Thermal Engineering and Environment: August 20-22, 2005; Corfu, Greece 2005 WSEAS Press 106 109

[B11] Huang HC Yin CP Ker YT Lin TF Hwang GJ, Chen CK Enhancement of boiling heat transfer in water through adding solid particles The 11th International Symposium on Transport Phenomena: November 29-December 3 1998; Hsinchu, Taiwan 1998 264 272

[B12] Grabar KC Freeman RG Hommer MB Natan MJ Preparation and characterization of Au colloid monolayers Anal Chem 1995 67 735 743 10.1021/ac00100a008

[B13] Cengel YA Heat Transfer: A Practical Approach 2003 McGraw Hill: Singapore

[B14] Tsai CY Chien HT Ding PP Chan B Luh TY Chen PH Effect of structural character of gold nanoparticles in nanofluid on heat pipe thermal performance Mater Lett 2004 58 1461 1465 10.1016/j.matlet.2003.10.009

